# Structural and Evolutionary Analysis of Proteins Endowed with a Nucleotidyltransferase, or Non-canonical Palm, Catalytic Domain

**DOI:** 10.1007/s00239-024-10207-7

**Published:** 2024-09-19

**Authors:** Rodrigo Jácome

**Affiliations:** Facultad de Ciencias, Universidad Nacional Autónoma de México, Mexico City, México

**Keywords:** Polymerase, Nucleotidyltransferase fold, Structure-based phylogeny, Deep evolutionary events, Structural evolution

## Abstract

Many polymerases and other proteins are endowed with a catalytic domain belonging to the nucleotidyltransferase fold, which has also been deemed the non-canonical palm domain, in which three conserved acidic residues coordinate two divalent metal ions. Tertiary structure-based evolutionary analyses provide valuable information when the phylogenetic signal contained in the primary structure is blurry or has been lost, as is the case with these proteins. Pairwise structural comparisons of proteins with a nucleotidyltransferase fold were performed in the PDBefold web server: the RMSD, the number of superimposed residues, and the Qscore were obtained. The structural alignment score (RMSD × 100/number of superimposed residues) and the 1-Qscore were calculated, and distance matrices were constructed, from which a dendogram and a phylogenetic network were drawn for each score. The dendograms and the phylogenetic networks display well-defined clades, reflecting high levels of structural conservation within each clade, not mirrored by primary sequence. The conserved structural core between all these proteins consists of the catalytic nucleotidyltransferase fold, which is surrounded by different functional domains. Hence, many of the clades include proteins that bind different substrates or partake in non-related functions. Enzymes endowed with a nucleotidyltransferase fold are present in all domains of life, and participate in essential cellular and viral functions, which suggests that this domain is very ancient. Despite the loss of evolutionary traces in their primary structure, tertiary structure-based analyses allow us to delve into the evolution and functional diversification of the NT fold.

**Supplementary Information:**

The online version contains supplementary material available at 10.1007/s00239-024-10207-7.

## Introduction

Polymerases partake in key cellular processes such as DNA replication, transcription, and several DNA repair pathways. As shown in Table 1, polymerases are versatile enzymes and come in a wide array of structural folds. In the early 90 s, when the elucidation of gene and protein sequences was beginning to gain momentum, DNA polymerases were classified into large families based on the presence of specific sequence motifs (Delarue et al. 1990; Ito and Braithwaite 1991). A, B, and C families were named according to their homology with the prototypic *Escherichia coli* genes; since eukaryotic pol β had no homologous gene in bacteria, its ortologs were classified as X-family (Ito and Braithwaite 1991). A few years later, a new family of replicative polymerases was identified in Archaea, and it was named D-family (Cann and Ishino 1999). Finally, formerly known as the UmuC/DinB/Rev1/Rad30 superfamily, low fidelity polymerases involved in repair pathways were unified under Y-Family (Ohmori et al 2001).

**Table 1:** Structural diversity, versatility, and biological distribution of RNA and DNA polymerases

Structural fold	Right-hand DNA & RNA polymerases			Nucleotidyltransferase fold			Double-Psi β-Barrel		
	A-Family DdDp	B-Family DdDp	Y-Family DdDp	C-Family	X-Family	Templ-Ind RNA pols	D-Family	DdRp	RdRp
Bacteria	Repair	Repair	Repair	Replication	Repair	RNA-mod		Transcription	
Archaea		Replication (Crenarchaeota)	Repair		Repair	RNA-mod	Replication	Transcription	
Eukaryotes	Mitochondrial replication	Replication	Repair		Repair	RNA-mod		Transcription	RNAi – plants

*DdDp* DNA-dependent DNA polymerase; *RdRp* RNA-dependent RNA polymerase; *RT* reverse transcriptase; *DdRp* DNA-dependent RNA polymerase; *Templ-Ind RNA pol* template-independent RNA polymerase; *RNA-mod* RNA modifying

**Fig. 1:**
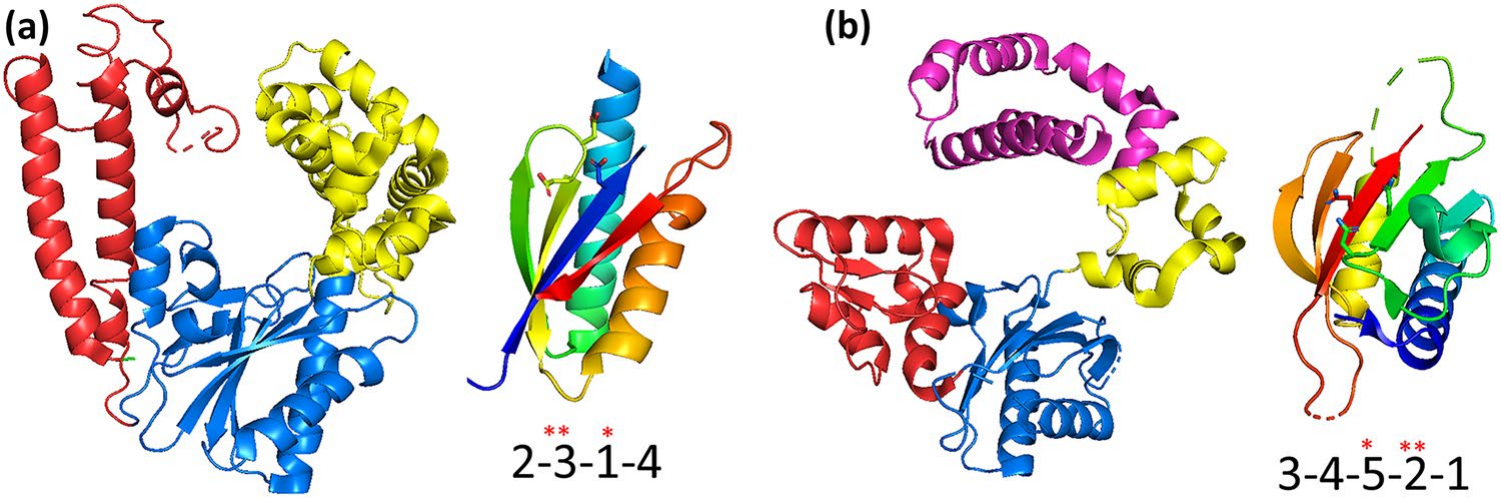
Comparison between the tertiary structures of right-hand polymerases and nucleotidyltransferases. **a** Depiction of right-hand polymerases and its corresponding canonical palm domain (Edited from PDB 1D8Y). **b** Depiction of a polymerase endowed with the nucleotidyltransferase fold and its corresponding non-canonical palm (Edited from PDB 4M9G). The polymerases domains are colored as follows: red—thumb; blue—palm; yellow—fingers; magenta—8 kDa. The canonical and non-canonical palm subdomains are colored following the rainbow spectrum with blue colors corresponding to the N-terminal residues and red colors to the C-terminal residues. The side chains of the catalytic aspartates are shown as sticks. The numbers below the palm domains correspond to the order in which the structures are in the sequence. The asterisks highlight the location of the catalytic aspartic acids

As tertiary structures of distinct polymerases were obtained, it became evident that many of them adopted a so-called right-hand configuration consisting of three functional domains, namely, palm, fingers, and thumb (Fig. 1a) (Joyce & Steitz 1994), the former being the catalytic domain in which two aspartic acid residues were universally conserved. Moreover, they all formed phosphodiester bonds assisted by two divalent metal ions coordinated by the acidic residues (Steitz 1999). The highly conserved palm domain, also known as the “canonical palm,” consists of a β-sheet formed by 4–5 strands, and two alpha helices “supporting” the sheet (Fig. 1a). As shown in Table 1, this fold is present in many polymerases including A-family, B-family, and Y-family DNA-dependent DNA polymerases (DdDp), viral DNA-dependent RNA polymerases (e.g., the T7 Phage DdRp), viral- and cellular RNA-dependent DNA polymerases (e.g., reverse transcriptases), and viral RNA-dependent RNA polymerases (RdRp), highlighting the versatility of this group of enzymes and their intricate evolutionary history (Mönttinen et al. 2016; Jácome et al. 2022).

The polβ nucleotidyltransferases (NTs) superfamily is an extremely diverse group of enzymes that includes X-family DdDps as well as many other nucleotidyltransferases (Holm and Sander 1995; Aravind and Koonin 1999; Kuchta et al. 2009). The overall reaction of these NTs is to transfer nucleotide monophosphates to hydroxyl groups (Kuchta et al. 2009). These enzymes share important structural and functional characteristics. They all display a common fold at their catalytic core consisting of a β-sheet formed by four to six strands and two α-helices at the back of the sheet (Fig. 1b). Furthermore, they all depend on the same mechanism of action, in which three universally conserved acidic residues coordinate two divalent metal ions to form phosphodiester bonds (Steitz 1999). To illustrate their functional versatility, these enzymes include DNA polymerases such as bacterial replicative DNA polymerases, and eukaryotic polymerase β, as well as RNA polymerases such as prokaryotic- and eukaryotic poly(A)-polymerases, tRNA CCA-adding enzymes, and kanamycin nucleotidyltransferase.

These structural and functional features are similar to the Superfamily of right-hand DNA- and RNA polymerases (Steitz 1999). However, as noted by Holm and Sander (1995), there are important differences between these enzymes. The connectivity of the palm subdomain’s structural elements is different; in the case of the right-hand polymerases, the palm domain strands' order is 2-3-1-4, whereas in the NTs the order is 3-4-5-2-1 (Fig. 1). Moreover, the location of the catalytic acidic residues also differs. The catalytic aspartic acids of the canonical palm are always located in strand #1 and in the loop connecting strands #2 and #3, while, in the non-canonical palm, two of them stand in strand #2, while the third conserved residue is on strand #5 (Fig. 1). Hence, the most possible scenario is that these polymerases are not homologous (Sawaya et al. 1994; Bailey et al. 2006).

Enzymes endowed with a NT fold are present in the three domains of life as well as in some dsDNA viruses; however, their distribution among the organisms is not homogeneous. For instance, based on their primary structures, the CCA-adding enzymes of bacteria and eukaryotes, key players for the correct maturation of functional tRNAs, were assigned to a different class compared to those of archaea (Yue et al. 1996); a fact that was corroborated with the visualization of their corresponding tertiary structures (Li et al. 2002; Augustin et al. 2003; Xiong et al. 2003).

Previous works classified nucleotidyltransferases based on the information available at the time of their publication. Yue et al. (1996) divided the NTs sequences in two main classes depending on the presence of sequence motifs around the active site. A few years later, using sequence profiles and the identification of unique signatures, Aravind and Koonin (1999) clustered the superfamily of NTs in 9 large groups, some of which could be subdivided in families. More recently, using remote homology detection tools, Kuchta et al. (2009) expanded and refined the previous classification schemes in 26 groups; 16 of these groups consisted of well-characterized proteins, whereas 10 groups included uncharacterized proteins with a putative NT fold and, due to the lack of some of the catalytic key residues, unknown biological functions. Except for the conserved catalytic residues, the level of sequence conservation between the different NTs is extremely low, even within the same group. None of these works included Family-C DdDps in their classification systems, since the similarity between other NTs and these DdDps is not detectable through primary structure-based approaches. Different works have demonstrated that structure-based phylogenies are a valuable alternative for the study of protein superfamilies with high levels of divergence and versatility, e.g., right-hand polymerases (Mönttinen et al. 2016; Jácome et al. 2022), proteases (Mönttinen et al 2019), and 3′-5′ exonucleases (Cruz-González et al. 2021). Thereof, we have built structure-based phylogenies and phylogenetic networks of the experimentally obtained NTs tertiary structures available, whose results we have complemented with biological and functional information, providing quite a complex evolutionary picture of these enzymes.

## Material and Methods

### Search for Structural Homologs and Structures Selection

A search for proteins’ structures endowed with a NT fold was performed in the PDBeFold web server (Krissinel and Henrick 2004) (https://www.ebi.ac.uk/msd-srv/ssm/) with default parameters using well-characterized proteins such as eukaryotic polymerase β (PDB 1BPB), eukaryotic poly(A) polymerase (PDB 1F5A), bacterial CCA-adding enzyme (PDB 1MIV), and bacterial Pol C (PDB 2HPI) as a starting point. The aim of these searches was to identify the maximum diversity in terms of the functions performed by these enzymes, and the biological diversity whence the structures were obtained. We discarded structures with a poor resolution, i.e., structures with a resolution above 4 Å. The same initial structures were used as queries in the Structome web server (Malik et al. 2023) to ensure that all the relevant structures (Qscore over 0.15) had been included in the dataset. Finally, a manual check of the Pfam families included in the Clan CL0260 NTP_transf (https://www.ebi.ac.uk/interpro/set/pfam/CL0260/) in the InterPro web server was performed, and structures that had not been previously identified through structural homology searches were also included. The complete set of proteins used in this study is displayed in Supplementary Table 1.

### Dendogram and Phylogenetic Network Construction

Pairwise comparisons between the selected tertiary structures were performed in the PDBeFold webserver with default parameters (Krissinel and Henrick 2004). The Qscore, the Root-mean-square deviation (RMSD), and the number of superimposed residues from each pairwise comparison were obtained. To normalize the results, we calculated two structural distance metrics: the structural alignment score (SAS) using the following formula (RMSD × 100)/Number of superimposed residues (Subbiah et al. 1993), and the 1-Qscore as in Malik et al. (2020), since this score also normalizes the results of the structural superpositions by pondering the RMSD, the number of superimposed residues, and the length of the compared proteins. A distance matrix was built for each score and used as input for the Fitch program included in the PhYLIP version 3.95 package. The resulting trees were visualized and edited in Figtree (http://tree.bio.ed.ac.uk/software/figtree/). As a support to the dendograms and to solve possible conflicting evolutionary signals, the Neighbor-Net algorithm (Bryant and Moulton 2004) included in the program Splitstree 4.0 (Huson and Bryant 2006) was used to calculate phylogenetic networks with the structural distance matrices as input.

### Tertiary Structures’ Edition and Depiction

All the figures containing tertiary structures were edited and depicted with the PyMol 2.4.1 software (The PyMOL Molecular Graphics System, Version 2.0 Schrödinger, LLC).

## Results and Discussion

Sixty-one experimentally obtained structures were selected for the structural comparisons, from which one belongs to archaea, 28 belong to bacteria, four belong to dsDNA viruses, and 28 belong to eukaryotes. Supplementary Table 1 depicts the characteristics of the selected structures.

**Fig. 2:**
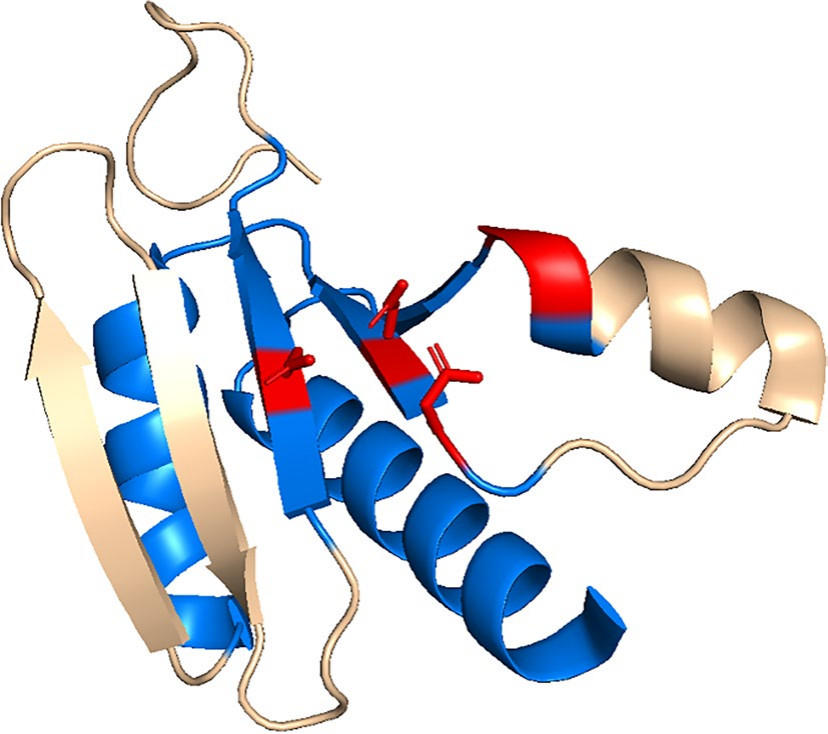
Depiction of the conserved catalytic NT fold. The regions colored in blue are conserved in all the structures compared in this work. The catalytic residues and the GS/GG NT signature motif are colored in red (Edited from PDB 5HC9)

Enzymes endowed with a nucleotidyltransferase fold participate in a wide array of cellular and viral functions. Previous works based on primary structure analyses had identified a defining sequence pattern in these enzymes: hG[GS]x(9,13)Dh[DE]h (Aravind and Koonin 1999). Overall, the structural pairwise comparisons of this work yield an average of 124.2 residues and an average SAS of 3.148. These conserved residues constitute the core of the NT fold (Fig. 2), and consist of three β-strands, corresponding to strands 1, 2, and 5 in Fig. 1b, and in which the catalytic acidic residues are located; a helix preceding strand 1 which is stacked below the β-sheet; the region connecting strands 1 and 2 in which one glycine is universally conserved; and a helix following strand 2.

**Fig. 3:**
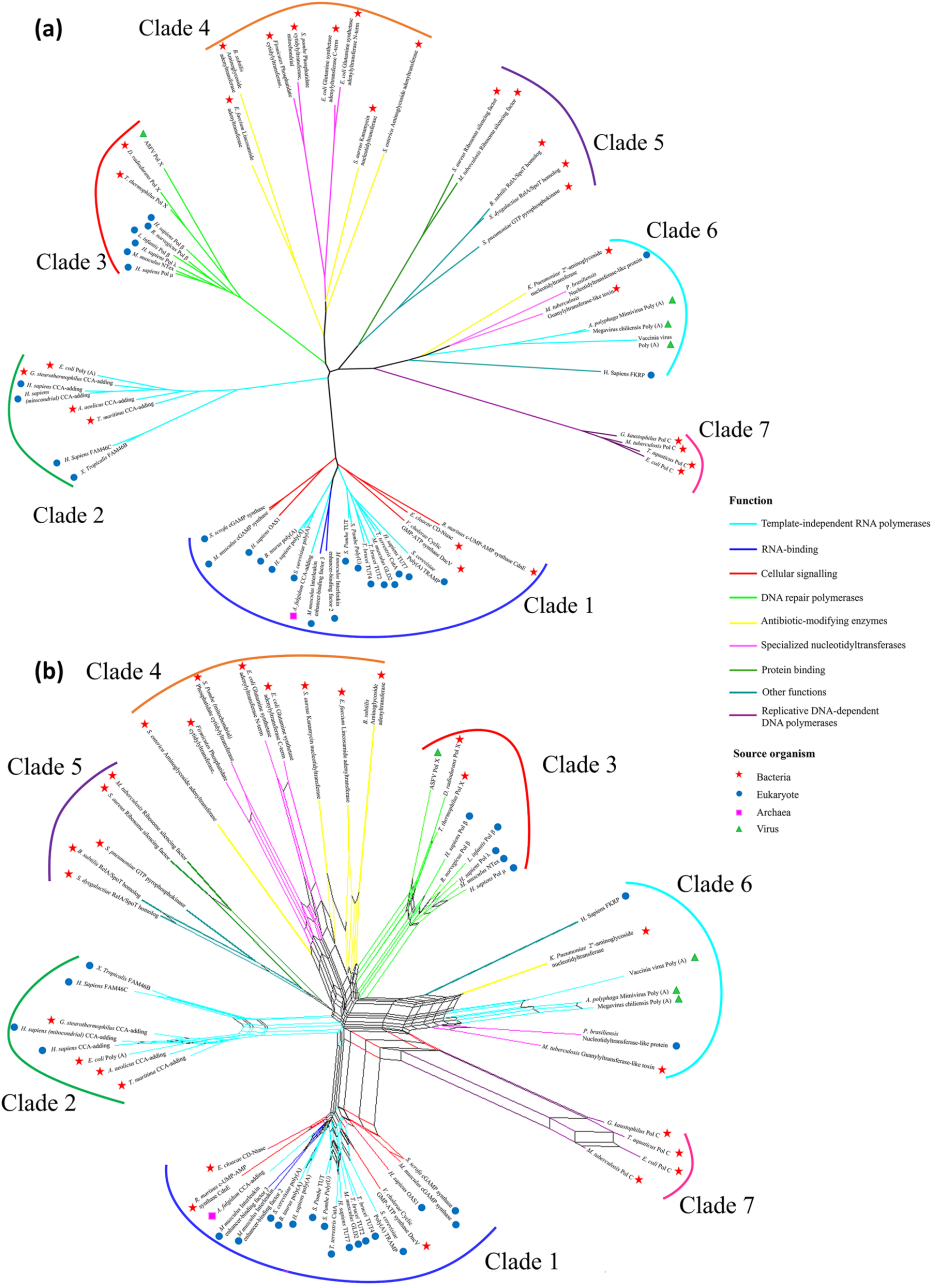
SAS-derived dendogram and phylogenetic network of proteins endowed with an NT fold. **a** Structure-based dendogram and **b** structure-based phylogenetic network of proteins endowed with an NT fold based on the Structural Alignment Score. The name of the organism that the structure was obtained from, and the name of the protein is written at each branch tip. The branches are colored according to the enzymatic function: cyan—template-independent RNA polymerases; blue—RNA binding; red—cellular signaling; chartreuse green—DNA repair polymerases; yellow—antibiotic-modifying enzymes; pink—specialized nucleotidyltransferases; olive green—protein binding; purple—replicative DNA-dependent DNA polymerases; turquoise blue—other functions. The symbol outside each enzyme corresponds to the cellular domain from which the structure was obtained: Bacteria—red star; Archaea—pink square; Eukaryotes—blue circle; Viruses—green triangle

One dendogram and one phylogenetic network were built for each of the two structural distance metrics, i.e., the SAS and the 1-Q score. The dendograms and the phylogenetic networks consistently display seven well-defined clades (Fig. 3 and Supplementary Fig. 1), and in most cases, the relationships within each clade are conserved. As will be described throughout the following sections, the evolutionary signal derived from the structural comparisons is supported by the presence of conserved additional domains, the biological distribution of the enzymes, and their function. The phylogenetic networks (Fig. 3b and Supplementary Fig. 1b) show a reticulate tree pattern, albeit there are some sections near the bases in which the evolutionary signals are not so sound. This “noise” is more evident in the SAS phylogenetic network, in which the base of some of the clades cannot be clearly distinguished. The results from the pairwise comparisons (RMSD, number of superimposed residues, SAS, and 1-Qscore) are included as Supplementary Table 2. A more detailed description and analysis of the resulting dendogram and the phylogenetic network are provided in the following sections.

### Clade 1: Eukaryotic RNA-Modifying Polymerases, Archaeal CCA-Adding Enzyme, Cyclic GMP, and AMP Synthases

**Fig. 4:**
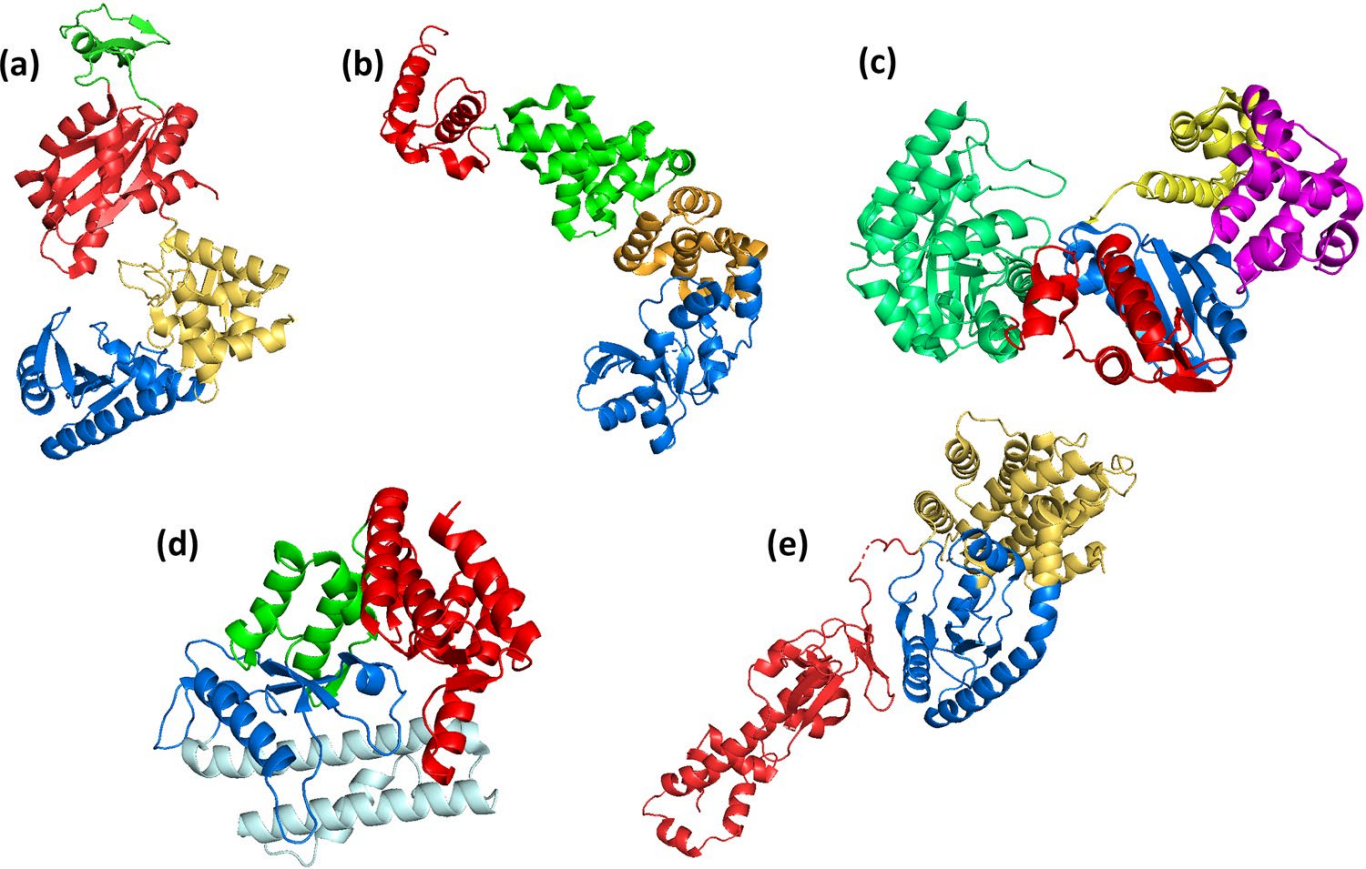
Visualization of representative structures of proteins endowed with the NT fold. **a** *Archaeoglobus fulgidus* CCA-adding enzyme (PDB 4X4P). Domains are colored as follows: blue—NT; gold—Central domain; red—RNA-binding domain; green—tail extension. **b** *Geobacillus stearothermophilus* CCA-adding enzyme (PDB 1MIV). Domains: blue—head domain; yellow—neck domain; green—body domain; red—tail/legs domain. **c** *Thermus thermophilus* HB8 PolX (PDB 3AU2). Domains: red—thumb; blue—palm; yellow—fingers; magenta—8 kDa; light green—PHP. **d** *Escherichia coli* Glutamine synthase C-terminal domain (PDB 3K7D). Domains: blue—NT; green—connection; red—C-terminal. **e** *Bacillus subtilis* RelA/SpoT homolog (PDB 6YXA). Domains: yellow—hydrolase domain; blue—synthetase domain; red—GTPase and Alpha helical domain

A first main clade (Clade 1 in Fig. 3 and Supplementary Fig. 1) is integrated almost entirely by RNA-modifying NTs, including the *Archaeoglobus fulgidus* CCA-adding enzyme, eukaryotic canonical- and non-canonical poly(A) polymerases, nuclear factors NF90 and NF45 (also named interleukin (IL) enhancer-binding factors 3 & 2, respectively), and eukaryotic- and bacterial cyclic GMP and AMP synthases as well as 2′-5′oligo adenylate synthase. Within this clade, the mean number of superimposed residues is 235 (mean SAS 1.178, mean 1-Q score 0.753). Besides the NT fold, all the enzymes of this clade display a central domain, which is mainly helical and carries the nucleotide-binding residues (Fig. 4a). In the case of PAPs and the archaeal CCA-adding enzyme, they possess an additional RNA-binding domain in the C-terminus (Fig. 4a). This domain displays the characteristic RNA-recognition motif (RRM) fold consisting of a 4-stranded β-sheet flanked by two helices (Xiong et al. 2003). In the case of the archaeal CCA-adding enzyme, the C-terminal domain is endowed with a “tail” extension that interacts with the tRNA (Fig. 4a) (Xiong and Steitz 2004).

From a functional perspective, even though most of the enzymes that form this clade modify some form of RNA, their substrate specificity, and the cellular processes in which they partake are extremely heterogeneous. Canonical PAPs are essential in the eukaryotic post-transcriptional maturation of mRNA by adding a tail of adenines to the 3’end, enhancing its stability within the nucleus; multiple copies of the canonical PAPs are present in eukaryotes (Martin et al. 2000; Yang et al. 2014). The CCA-adding enzyme is also crucial for the post-transcriptional processing and maturation of tRNA in cells (Xiong et al. 2003); notably, as mentioned above, at least two versions of CCA-adding enzymes endowed with the NT fold exist, one in archaea, and the second one in bacteria and eukaryotes. As the dendograms show, these enzymes have undergone a large process of functional diversification in eukaryotes. Non-canonical PAPs are ubiquitous in eukaryotes and are also involved in key steps of RNA processing. Among non-canonical PAPs, poly (U) polymerases and terminal uridylyl transferases (TUT) have been shown to play important roles. For instance, the addition of uridines leads to mRNA decay and degradation during germline development, embryogenesis, cellular differentiation (Zigáčková and Vaňáčová 2018). TUT4/7, either by monouridylation or oligouridylation, also marks non-coding RNAs for degradation including different forms of miRNAs and snRNAs (Zigáčková and Vaňáčová 2018). Conversely, uridylation is essential in the stabilization of other RNAs such as U6 snRNAs or the mitochondrial gRNA and mRNA processing of parasitic protists, e.g., Trypanosoma brucei (Rajappa-Titu et al. 2016). NF90 and NF45 dimerize and form a complex which have been associated with the regulation of eukaryotic gene expression by its binding to different RNA substrates (Wolkowicz and Cook 2012). The catalytic aspartic acids are not present in either of these two structures, indicating that they no longer carry out the transferase activity (Wolkowicz and Cook 2012). Finally, this clade includes signaling proteins that participate in bacterial and animal immune responses (Govande et al. 2021). It must be highlighted that in the SAS-derived dendogram and the phylogenetic network, these proteins are in two branches, one corresponding to the bacterial and the other corresponding to eukaryotic proteins, whereas in the 1-Q-derived constructions, the six enzymes form one single clade. This might be due to the conformational changes elicited by the substrates to which the enzymes were bound during the crystallization process, e.g., double-stranded RNA or DNA. In the case of mammalian cGAMP synthases, the binding of bacterial or viral dsDNA enhances the synthesis of cyclic GMP-AMP (Civril et al. 2013). On the other hand, oligoadenylate synthase binds to dsRNA and synthesizes 2′-5′,-linked iso-RNA. These two molecules trigger the IFN response (Donovan et al. 2013). Bacterial CD-NTases, e.g., DncV and CdnE, participate in the cyclic oligonucleotide-based antiphage signaling system (CBASS), which has been identified in Bacteria and Archaea (Kato et al. 2015; Duncan-Lowey and Kranzusch 2022). These enzymes sense phage DNA and synthesize second messengers such as 3′3′-c-UMP-AMP, 3′3′-cGAMP, or cyclic trinucleotides (Whiteley et al. 2019). Previous works have posited that the animal enzymes were recruited from prokaryotes via HGT (Kranzusch 2019; Morehouse et al. 2020; Patel et al. 2022).

Even though immune-related enzymes and template-independent RNA-modifying enzymes have a conserved architecture, they display two completely different modes of substrate binding. As shown in Supplementary Fig. 2, in RNA-modifying enzymes such as the archaeal CCA-adding pol, the binding surface is located on top of the active site, whereas the substrate-binding site of immune-related enzymes is located below the active site. As previously noted, (Torralba et al 2008) despite the functional differences between all the enzymes included in this clade, there is an evolutionary linkage between them, which can only be ascertained when their structures are compared. The presence of the archaeal CCA-adding enzyme and the prokaryotic CD-NTases might be a good indicator of their ancestry (Torralba et al. 2008).

### Clade 2: Bacterial/Eukaryotic CCA-Adding Enzymes and Bacterial Poly(A) Polymerases

A second well-defined clade in the dendograms and the phylogenetic networks (Clade 2 in Fig. 3 and Suppl. Figure 1) groups in one branch bacterial/eukaryotic CCA-adding enzymes and bacterial poly(A) polymerase, and eukaryotic non-canonical poly(A) polymerases FAM46B and FAM46C in another. The mean number of superimposed resides within this clade was 245, with a mean SAS of 1.376, and a mean 1-Q score of 0.756.

Bacterial/eukaryotic CCA-adding enzymes and bacterial poly(A) polymerases have been likened to a seahorse or a sea-otter, in which four domains were identified: head, neck, body and tail/legs (Fig. 4b) (Li et al. 2002; Toh et al. 2009). The active site of the enzymes is located in the head domain. The β-sheet of these enzymes’ NT fold has two additional strands compared to other NTs in the order 3-4-5-2-1-6-7; these strands link the head to the neck domain. The neck, body, and tail domains are formed by helices and interact with the T- and D-loops of tRNA (Fig. 4b). The bacterial PAP is also endowed with a head, neck, and body domains but lacks the tail extension, instead it has a less protruding small domain which has been named the “legs” domain (Toh et al. 2011). The conservation between CCA-adding enzymes and the bacterial PAPs extends beyond the head and neck domains. Even though the length of the helices that form the body domains is clearly different in the two types of enzymes, their connectivity and their direction are preserved, underscoring their evolutionary closeness (Toh et al. 2011). The structures of FAM46B and FAM46C are also endowed with the catalytic head and a helical neck domain; however, the body and the leg domains are absent.

CCA-adding enzymes are template-independent polymerases that specifically add the CCA triplet to the 3’-end of tRNAs post-transcriptionally, which is essential in cells for its amino-acid binding and its interaction with the ribosome (Tomita et al. 2004). Two similar CCA-adding mechanisms have been described in Bacteria (Tomita et al. 2004; Jones 2019). In genus such as *Geobacillus* and *Thermotoga*, one single enzyme adds the three nucleotides; whereas, in the Aquificae, Thermosdesulfobacteria, Deinococcus, Cyanobacteria, some Firmicutes and Gamma-Proteobacteria, one enzyme adds the two Cs, and a separate enzyme adds the terminal A (Tomita et al. 2004; Jones 2019). Phylogenetic analyses indicate that these enzymes share a common ancestor (Jones 2019). Interestingly, the same CCA-adding enzyme is employed to mark unstable tRNAs or anomalous RNAs by adding CCACCA for their degradation (Wilusz et al. 2011). Contrary to the poly(A) extension in eukaryotes, which is essential for mRNA maturation, the addition of the poly(A) tail to the 3′OH end of mRNA in bacteria serves as a marker for its degradation. Homologous PAPs to the CCA-adding enzymes have been only detected in the Beta, Gamma and Delta Proteobacteria (Jones 2019). The evolutionary scenario depicted by Jones (2019) posits that the ancestor of β and γ proteobacteria was endowed with a CCA-adding enzyme; a subsequent event of gene duplication and further mutations would eventually lead to the emergence of the PAP. On the other hand, FAM46B and FAM46C are highly specialized eukaryotic cytoplasmic poly(A) polymerases, whose functions have been associated with early embryo development (Hu et al 2020), and cell proliferation and sperm development (Chen et al 2020), respectively.

This clade displays a similar evolutionary scenario as Clade 1, in which bacterial enzymes could have emerged first, e.g., CCA-adding enzyme, followed by their horizontal transfer to eukaryotic cells, subsequently acquiring additional functions and different degrees of specialization in the latter.

### Clade 3: X-Family DNA-Dependent DNA Polymerases

A third main clade (Clade 3 in Fig. 3 and Suppl. Fig. 1) groups X-family DNA-dependent DNA polymerases. The overall pairwise comparisons of these polymerases yielded an average of 213 superimposed residues (mean SAS 1.219, mean 1-Q score 0.712). Within this clade, one branch includes the bacterial- and the African swine fever virus pols X, whereas the other branch groups eukaryotic polymerase λ, polymerase μ, DNA nucleotidyltransferase, and eukaryotic polymerases β. In the 1-Q-derived results, the sole difference is that the ASFV polX is closer to the eukaryotic pols. These polymerases adopt a ring-like structure consisting of two functional domains, a N-term deoxyribose phosphate (dRP) lyase domain, also known as the 8 kDa domain, and the C-term polymerase domain, in which fingers, palm, and thumb subdomains were also identified (Fig. 4c) (Batra et al. 2006). In the case of bacterial and archaeal polXs, their spatial configuration is different. The 8 kDa is in an extended position and not enclosing the active site. Moreover, these PolXs are endowed with a polymerase and histidinol phosphatase (PHP) domain in the C-terminus (Fig. 4c), forming a back wall to the polymerase domain (Nakane et al. 2009; Rodríguez et al. 2019).

X-family polymerases are universally distributed, and their participation in DNA repair processes filling nucleotide gaps has been reported in the three domains of life (Moon et al. 2017). Unlike all the polymerases discussed so far, which are RNA polymerases, these enzymes are DNA-dependent DNA polymerases with a preference for single-nucleotide gaps as their substrate (Mejía et al. 2014). Eukaryotic polymerases μ, λ, and TdT participate in V(D)J recombination during immunoglobulin and T-cell receptors formation (Loc’h and Delarue 2018). Previous works showed that PolX homologs could only be identified in 13% of the available bacterial whole genomes and 31% of the archaea; moreover, 90% of the archaeal enzymes belong to the Euryarchaeota phylum (Prostova et al. 2022). Phylogenetic analyses had previously posited that polX emerged in Gram-positive Bacillus bacteria and duplication events would lead to two branches in eukaryotes, one encompassing pol β and pol λ, and another encompassing pol μ and TdT (Bienstock et al. 2014). The diversity and versatility of PolXs in eukaryotes also depict a functional explosion of the enzymes following their emergence.

PolX has also been identified in the African swine fever virus as well as in other dsDNA virus belonging to the so-called giant viruses (Nucleocytoviricota phyla) such as Megavirus, Mimivirus, and certain phycodnaviruses (Chen et al. 2017; Lad et al. 2023). During viral replication within swine macrophages, the ASFV is counterattacked by the cellular defense mechanisms, which leads to an increase in the viral mutation rate. The expression of viral PolX together with an AP endonuclease has proven to diminish the mutation rate, favoring viral genomic stability (Redrejo-Rodríguez et al. 2013). This viral enzyme has been described as a minimal version of PolXs consisting only of the NT fold and the fingers' subdomain (Maciejewski et al. 2001; Wu et al. 2014). On the other hand, PolXs identified in other giant viruses seem to be endowed with most of the essential catalytic and nucleotide-binding residues as well as the complete set of domains: palm, fingers, thumb, and 8 kDa (Redrejo-Rodríguez and Salas 2014). Supplementary Fig. 3 shows a homology-based model of *A. polyphaga mimivirus* PolX displaying the characteristic ring-shape figure of these proteins. It is widely accepted that the genomes of these large DNA viruses are dynamic and have been incorporating and losing genes throughout their evolutionary pathways (Filée 2015; Campillo-Balderas et al. 2023). Therefore, it is not surprising to find proteins such as X-family polymerases in some of the families of this heterogeneous group of viruses.

### Clade 4: Specialized Bacterial Nucleotidyltransferases and Antibiotic-Modifying Enzymes

A fourth clade groups antibiotic-modifying enzymes as well as specialized nucleotidyltransferases (Clade 4 in Fig. 3 and Suppl. Fig. 1). As seen in Fig. 3, SAS-derived distances group antibiotic-modifying enzymes in two branches, one includes *B. subtilis* aminoglycoside 6-adenyltransferase and *E. faecium* lincosamide adenylyltransferase, whereas the second includes *S. enterica* aminoglycoside adenyltransferase and *S. aureus* kanamycin NT. The two-glutamine synthase (GS) adenylyltransferase domains and the phosphatidate cytidylyltransferases form another branch located between the two formers. In the 1-Q-derived distances, all the antibiotic-modifying enzymes are clustered in a single branch, and the other enzymes are clustered in another. The results of the pairwise comparisons for this clade yield an average of 137.6 superimposed residues, with an average SAS of 3.062, and a 1-Q mean of 0.908. The core of these enzymes consists of two domains, an N-terminal nucleotidyltransferase catalytic domain, followed by a helical C-terminal domain (Fig. 4d). All these enzymes display a series of conserved structural elements following the NT-catalytic domain’s strand #5. These elements consist of a helix pointing toward the C-term domain, followed by a β-strand that adds into the NT-catalytic β-sheet, and a second helix that “ascends” toward the C-term helical domain (Fig. 4d). Compared to the other enzymes of this branch, the GS adenylyltransferases are larger structures. The catalytic domain is “wrapped” by the longer helices of the C-terminal domain, which extend downwards and interact with the helices preceding the NT fold, and which are stacked below it.

Glutamine synthetase is a key enzyme in bacterial nitrogen metabolism, partaking in ammonia assimilation through the ATP-dependent synthesis of glutamine from glutamate (Xu et al. 2010). The adenylyltransferase enzyme is a bifunctional protein that adds AMP or removes AMP from each GS monomer. The adenylyltransferase is composed of two homologous domains endowed with NT folds, each of which carries out opposite chemical reactions|, and connected by a so-called regulatory domain (Xu et al. 2004, 2010). The N-terminal domain deadenylylates GS by phosphorolysis leading to its activation, whereas the C-terminal domain inactivates it by adding an AMP (taken from ATP) to a tyrosine in the GS (Xu et al. 2010; Van Heeswijk et al. 2013). Previous analyses showed a high level of structural conservation between these domains, and that several residues in the active site and the substrate-binding moieties are also conserved, suggesting that one of the domains arose by gene duplication (Xu et al. 2010). On the other hand, the translocator assembly and maintenance 41 (Tam41) have been mainly identified in eukaryotes, associated with the generation of cardiolipin in the mitochondrial membrane. (Kimura et al. 2022). Tam41 removes one CMP from CTP and transfers it to PA in the mitochondrial inner membrane, forming CDP-DAG, which is an intermediate in the biosynthesis of cardiolipin (Jiao et al 2019). Tam41 homologous proteins have also been identifiedin bacteria such as firmicutes and proteobacteria (Kimura et al. 2022). Structural and biochemical studies have shown that the bacterial version of Tam41 can also synthesize CDP-DAG, probably binding to the lipid membrane by residues of its C-terminal domain (Kimura et al. 2022). These works have also revealed that only one of the three characteristic acidic residues of the NT fold are present in Tam41’s active site, and positively charged residues accommodate CTPin a different position within the active site cavity (Jiao et al. 2019; Kimura et al. 2022).

Various nucleotidyltransferases that chemically inactivate aminoglycosides and lincosamides have been identified in pathogenic bacteria, as well as in mobile genetic elements such as plasmids, integrons, or transposons (Ramírez and Tolmasky 2010). Like the GS adenylyltransferase C-terminal domain, these enzymes add an ATP-derived AMP group to one of the antibiotic’s hydroxyl moieties hindering their binding to the ribosome. It has been posited that antibiotic-modifying enzymes are evolutionarily related to other enzyme superfamilies, usually housekeeping proteins (Morar and Wright 2010). As described above, all the enzymes from this clade perform similar functions, which added to their structural similarity support their evolutionary relatedness. It is also possible that some ANTs and Lin proteins might have diverged from GS adenylyltransferases, which are broadly distributed in bacteria and play an essential role in nitrogen metabolism.

### Clade 5: Bacterial RelA/SpoT-Like Homologs and Ribosome Silencing Factors

The fifth conserved clade groups proteins belonging to the RelA/SpoT-like homologs in one branch, and ribosome silencing factors in a second one (Clade 5 in Fig. 3 and Suppl. Fig. 1). The enzymes included in this clade do not carry out nucleotidyltransferase reactions; instead, they are highly specialized versions of the NT fold, involved in critical steps during bacterial stress responses. The pairwise comparisons yield an average of 116.4 superimposed residues (mean SAS 2.334, mean 1-Q 0.7768) for the structures included in this clade. Although in the SAS- and the 1-Q analyses they form one branch, the relation with respect to the other clades cannot be clearly ascertained. Bacterial RelA/SpoT are large proteins, in which the N-terminal domain carries out hydrolase and synthetase activities, whereas the C-terminal serves as a regulatory domain (Fig. 4e) (Pausch et al. 2020). The active site of the synthetase domain is slightly different from previously described NT folds (Supplementary Fig. 4) as it lacks one of the three conserved acidic residues, and the GS/GG motif is replaced by GR. Moreover, the region following strand 1 is longer compared, forming a positively charged “wall” next to the active site, which interacts with the phosphates of a donor ATP (Hogg et al. 2004). A long loop protrudes upwards between strands 3 and 4, in which conserved residues interact with the acceptor GDP (Hogg et al. 2004). Ribosome silencing factors are also endowed with the NT fold; however, they lack the essential catalytic residues, hence, they do not carry out a nucleotidyltransferase reaction. Compared to other NT folds described in this work, most of which display variations in the number of strands, the enzymes pertaining to this clade all have a 5-stranded β-sheet, with a longer and bulkier structure following strand #1.

When faced with adverse conditions such as starvation and temperature or pH changes, cells activate stress responses that promote their survival including the synthesis of alarmones (Driller et al. 2023). Bacterial bifunctional RelA/SpoT homologs participate in the synthesis and hydrolysis of the alarmone (p)ppGpp, which is crucial in the case of amino-acid scarcity (Mechold et al. 2002; Hogg et al. 2004). The synthetase domain displays the main structural features of the NT fold, though it carries out a different chemical reaction, in which a pyrophosphate group from ATP is transferred to a GDP/GTP 3′OH releasing AMP when stalled ribosomes and faulty tRNAs accumulate in the cell (Hogg et al. 2004; Pausch et al. 2020). Many different enzymes that synthesize (p)ppGpp have been identified in bacteria and other eukaryotes including stand-alone variants; however, the most prevalent and the best distributed among bacteria corresponds to the bifunctional RelA/SpoT protein (Atkinson et al. 2011), probably indicating its ancient origins. It must be underlined that neither members of this RelA/SpoT homologs Superfamily nor the alarmone ppGpp have been identified in Archaea (Van der Does et al. 2023). As aforementioned, ribosome silencing factors lack the essential catalytic residues; instead, they bind to ribosomal protein L14 and prevent the formation of the 70S ribosomal complex, which, in turn, stalls protein synthesis during periods of starvation or during the stationary phase (Li et al. 2015; Khusainov et al. 2020).

### Clade 6

The sixth main clade (Clade 6 in Fig. 3 and Suppl. Fig. 1) includes a heterogeneous group of proteins endowed with the NT fold. These structures include dsDNA viral poly(A) polymerases, eukaryotic fukutin-related protein (FKRP), specialized nucleotidyltransferases, and a bacterial antibiotic-modifying enzyme. This clade is conserved in the SAS-and 1-Q-derived analyses, but its relationship to the other clades is not consistent: in the former it is closer to bacterial replicative DdDps, whereas in the latter, it is related to clade 2 (dendogram) or to clade 4 (phylogenetic network). The pairwise comparisons of these structures yield an average of 156.8 superimposed residues, a mean SAS of 2.260, and a mean 1-Q of 0.872. All these enzymes are endowed with the key catalytic residues and a slight variation of the conserved architecture of the NT fold. The β-sheet is wider and formed by 7 strands in the order 3-4-5-2-1-7-6 (two of the catalytic acidic residues in strand 2, and the third catalytic residue in strand 5); moreover, strand 7 is followed by two to three antiparallel helices located above the active site. FKRP is also endowed with a stem domain, similar to GalNAc transferase, N-terminal to the NT fold. Viral poly(A) polymerases are characterized by an N-terminal NT domain harboring the catalytic residues, followed by a C-terminal non-functional NT fold that may have originated after a duplication event (Supplementary Fig. 5) (Priet et al. 2015). Another distinctive feature of viral NTs is that their NT fold β-sheets are complemented by one or two β-strands that descend from the C-terminal residues next to strand 3.

Several mechanisms of enzymatic antibiotic inactivation have been described in bacteria (Cox et al. 2015). *K. pneumoniae’s* ANT(2″)-Ia inactivates aminoglycosides by transferring an AMP moiety to its 2″-OH, whereas the enzymes pertaining to clade 4 add the AMP to positions 4 or 6 (Cox et al. 2015). As shown by the fact that *K. pneumoniae’s* ANT(2″)-Ia is in a different clade, this work supports the notion that similar antibiotic-modifying enzymes might have emerged in several occasions. Secondly, the *M. tuberculosis* TgIT has been described as a high affinity guanylyltransferase, belonging to a type IV atypical toxin antitoxin system, although the exact mechanisms of interference with protein translation have not been yet elucidated (Yu et al. 2020). Similarly, structural studies of *P. brasiliensis* nucleotidyltransferase-like protein established its higher affinity for ATP and CTP; however, a precise function could not be determined (Coitinho et al. 2019). Finally, FKRP participates in the synthesis of α-dystroglycan, which is essential in the eukaryotic basement membrane. Interestingly, even though FKRP displays the characteristic residues of the NT fold, it performs a different reaction in which a CMP is released from cytidine diphosphate-ribitol (CDP-Rbo), and the ribitol-phosphate (RboP) moiety is transferred to an OH acceptor (Kuwabara et al. 2020). dsDNA viruses of the *Poxviridae, Asfarviridae, Mimiviridae, Megaviridae* and other unclassified giant viruses, all of which replicate in the cellular cytoplasm, encode several mRNA-modifying enzymes including poly(A) polymerases (Rodríguez and Salas 2013; Priet et al. 2015). In association with VP39, a processivity factor, the vaccinia virus PAP can extend the poly(A) tail hundreds of nucleotides (Gershon and Moss 1993; Moure et al. 2006). On the other hand, the mega-and mimivirales PAPs form homodimers, which, in turn, lead to their self-processivity (Priet et al 2015). The diversity of the functions in which this clade’s enzymes participate provide support to the notion that a small number of mutations are sufficient to bestow a protein with different enzymatic capabilities, which is also evident in other clades.

### Clade 7: Bacterial C-family DNA-Dependent DNA Polymerases

**Fig. 5:**
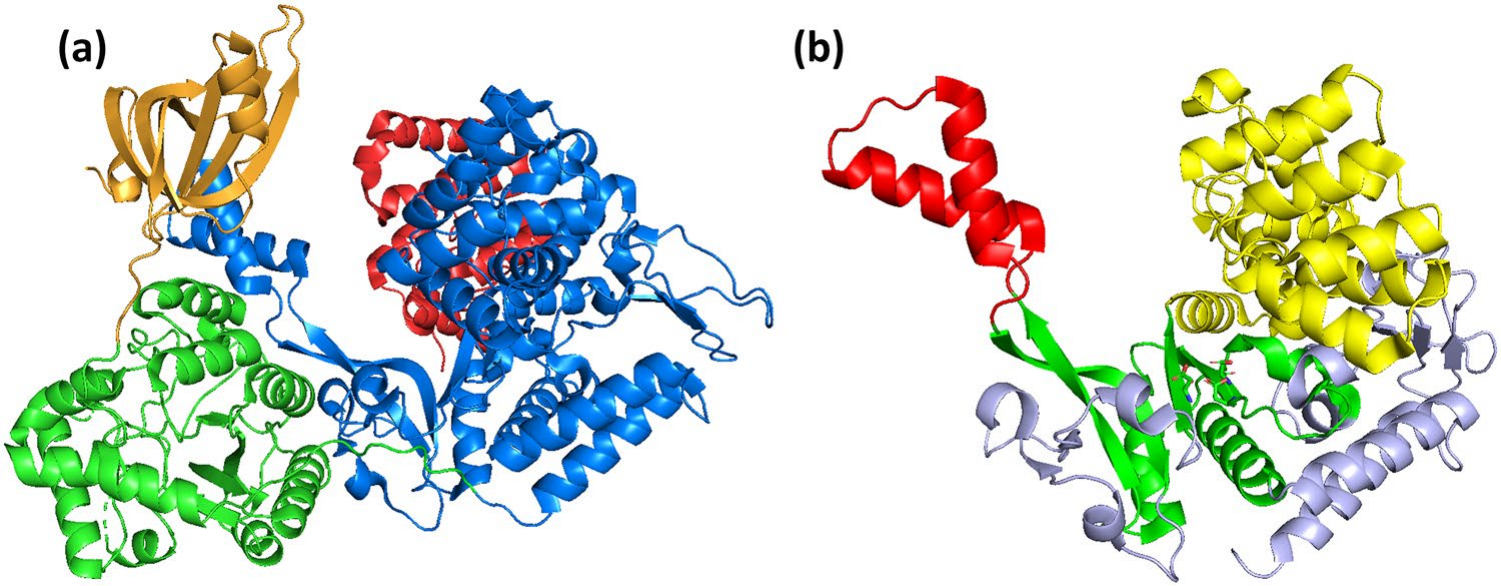
Representation of a C-family DNA-dependent DNA polymerase **a** *Geobacillus kaustophilus* C-family DNA-dependent DNA polymerase (PDB 3F2C); from the N-terminus to the C-terminus, the domains are colored as follows: gold—OB; green—PHP; blue—polymerase; red—DB. The exonuclease domain is not visible in the structure but is located between the PHP and the polymerase domains. **b** The domains of the polymerase domain: red—thumb, green—palm, yellow—fingers. The structural elements inserted in the NT fold are colored in gray (Edited from PDB 3F2C)

Bacterial replicative DdDps are all clustered in a single clade (Clade 7 in Fig. 3 and Suppl. Fig. 1). Within this clade, the pairwise comparisons yield an average of 475.6 superimposed residues, a mean SAS of 0.577, and a mean 1-Q of 0.713. Bacteria replicate their genomes through C-family polymerases, DnaE in Gram-negative bacteria, and PolC in Gram-positive bacteria (Evans et al 2008). These polymerases are large protein complexes in which distinct domains have been identified, including an Oligonucleotide-binding (OB) fold domain, a polymerase and histidinol phosphatase (PHP) domain, and the polymerase domain (Fig. 5a) (Bailey et al. 2006; Lamers et al. 2006; Evans et al. 2008). The polymerase domain is in turn subdivided in palm, fingers, and thumb subdomains (Fig. 5b), whose interactions with the nucleic acids are analogous to those of right-hand polymerases. The crystal structures of bacterial replicative polymerases (Bailey et al. 2006; Lamers et al. 2006; Evans et al. 2008) revealed that these enzymes are endowed with a NT fold. However, these are the only enzymes in which several structural insertions break the continuity of the “classical” NT fold. Between the GS motif and strand #2, approximately 40 residues form a pseudo-helical bundle below the fingers (Fig. 5b). In the case of the *E. coli* pol C, this extension is longer and forms a three-stranded β-sheet that projects outwards beyond the fingers. There is also an insertion between strands 3 and 4 forming several helices that extend upwards and outwards from the active site and interact with the primer strand, hence, the “thumb.” This insertion “pulls” strands 3 and 4, widening the active site cavity allowing for a dsDNA to fit. Finally, there is another insertion of approximately 40 residues between strands 4 and 5, forming two antiparallel β-strands, extending the β-sheet of the NT fold. Despite the presence of the conserved structural elements and motifs of the NT fold, the numerous differences explain the divergence to other proteins included in this work.

### Can the PHP Domain Give Us More Clues About the Evolution of These Enzymes?

**Fig. 6:**
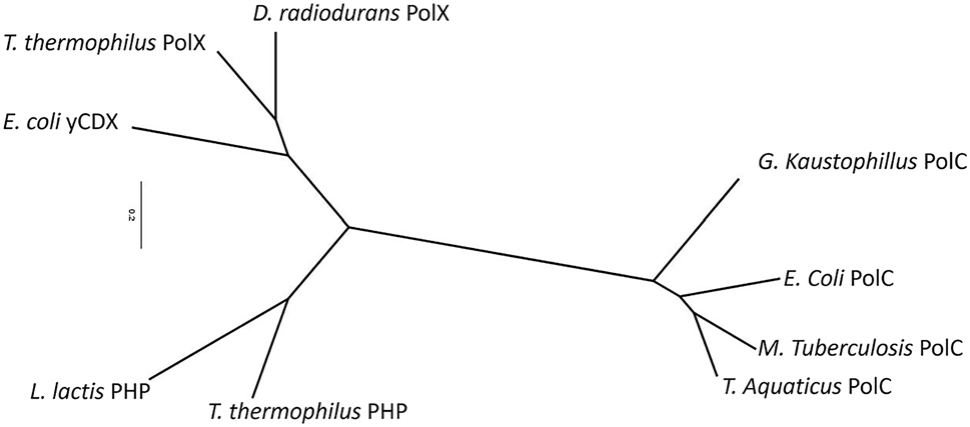
Structure-based dendogram of the PHP domains found in C-family DNA-dependent DNA polymerases and stand-alone PHP enzymes

Interestingly, certain bacterial enzymes with an NT fold are associated with PHP domains, probably hinting to a closer evolutionary link. However, different facts indicate that the enzymes acquired the domain at least on two independent occasions. First, the PHP domain is located N-terminal to the polymerase domain in replicative pols, whereas in X-family pols, the PHP domain is C-terminal. Furthermore, a dendogram based on structural comparisons (Fig. 6) between the PHP domains present in these pols, to which other stand-alone PHP enzymes were added, supports the hypothesis of multiple-domain acquisitions. Figure 6 shows that all the PHPs associated with replicative polymerases are clustered in a single branch, while those belonging to bacterial Pols X are closer to *E. coli’s* YcdX (Teplyakov et al. 2003) and to a branch grouping *Lactobacillus lactis* histidinol phosphate phosphatase (PDB 4GYF) and *T. thermophilus* monofunctional PHP (Omi et al. 2007).

### Thus, How Old is the NT Fold?

Previous works (Aravind and Koonin 1999) had posited that the NT fold was already present in the last common ancestor. The identification of enzymes endowed with the NT fold in the prokaryotic domains and their relevance in essential housekeeping processes, e.g., PolX and CCA-adding enzyme, supports this idea. Furthermore, bacteria seem to be the ones which have more thoroughly explored the functional capabilities of this fold. Considering the biological distribution and the processes in which the NT-endowed enzymes partake, we must assume that replicative bacterial polymerases, i.e., C-family pols, represent an ancient version of the NT fold, which is reflected in its highly divergent version of it.

Essential RNA modifications such as the addition of CCA to tRNAs might have emerged first followed by other important, albeit non-essential, processes like immunity or antibiotic inactivation. Moreover, the dendograms and the phylogenetic networks show that different levels of functional diversification and specialization might have arisen progressively. In the case of bacteria, enzymes endowed with the NT fold participate in many different essential pathways including DNA repair and nutrients assimilation, as well as in adaptive responses such as alarmone synthesis, or antibiotic modifications. Whereas in the case of eukaryotes, the NT fold seems to have undergone different degrees of specialization, exemplified by the identification of a single X-family representative in bacteria compared to the presence of up to four X-family homologs in several eukaryotes, or the wide array of eukaryotic RNA-modifying enzymes such as terminal uridyltransferases and specialized poly(A) pols. Throughout evolution, several enzymes with an NT fold have gained new functions through secondary losses of the catalytic residues, hence losing the nucleotidyltransferase activity. The scattered distribution of many of these enzymes plus the assumption that DNA replication was already settled in bacteria hint to the fact that the evolutionary processes depicted here are not so ancient, namely after the emergence of prokaryotes.

### The NT Fold, One Versatile Solution to Many Different Biological Riddles

Most of the proteins that assemble the cellular replisomes are clearly not homologous (Georgescu et al. 2015), including the replicative polymerases, which are different in each one of the cellular domains: C-family DdDps replicate bacteria, D-family DdDps replicate Archaea, B-family DdDps replicate eukaryotes, and Crenarchaeota. Apart from the presence of acidic residues coordinating divalent metal ions in their active site, the three DdDps display different structural folds, clearly highlighting a case of convergent evolution, i.e., three different polymerases evolved independently and replicate the DNA.

However, the biological distribution and the array of functions of the proteins shown in Table 1 clearly indicate that the cores of the three structural folds, i.e., the canonical palm domain of Right-hand pols, the NT fold, and the catalytic center of Double-Psi β-Barrel pols, were already present in the LUCA. It is possible that the ancient versions of these folds were non-specific in terms of preferred substrate and synthesized product, progressively acquiring specificity and specialization through adaptations, other domains’ accretions, duplication events, and lateral gene transfer. The versatility of the enzymes endowed with the NT fold clearly illustrates this point. Their functional diversity is mainly provided by the acquisition of additional domains, which has significantly broadened the number of possible substrates, considering that the end-product of the NT chemical reaction is frequently the same, i.e., the addition of nucleotides to a 3′OH. As shown throughout this work, two proteins endowed with the same domains (combined with certain key mutations) may carry out different functions, e.g., eukaryotic RNA-modifying enzymes and proteins involved in cellular signaling. As is the case with other (probably) very ancient enzymes that interact with nucleic acids such as exonucleases or right-hand polymerases, proteins endowed with a NT fold are far from being specific.

The results and the clusters presented in this work are similar to the classification proposed by Kuchta et al. (2009); however, the aim of this work is not to propose or modify their classification system, but to depict possible evolutionary scenarios. Based on this work’s results, there is a direct evolutionary relationship between bacterial glutamine synthetases and certain aminoglycoside-and lincosamide modifying enzymes, confirming Morar and Wright’s hypothesis (2010) about the origin of antibiotic-modifying enzymes from housekeeping genes. On the other hand, the evolutionary hypothesis presented here indicates that the NCLDVs probably “took” both their poly(A) pol as well as PolXs from cells, which is not surprising since these viruses have a very intricate evolutionary history adorned by multiple lateral gene transfers (Filée 2015). A paramount difference between previous works and this article is that the methodology employed here allowed the incorporation of C-family polymerases into the evolutionary scenario of proteins endowed with NT folds; however, it was not possible to assign polarity into the tree, preventing us to posit a more precise timeframe into the evolutionary events depicted here.

Despite being a very useful and powerful methodology, the evolutionary analysis derived from the comparison of proteins’ tertiary structures is far from being flawless. Different structural scores can be used to infer evolutionary distances. However, previous works have shown that normalized scores that incorporate not only the RMSD of a structural comparison, but also the length of the alignment and the length of the proteins, e.g., SAS, Qscore, or the TM-score, yield more accurate results (Zhang and Skolnick 2004; Malik et al. 2020; Jácome et al. 2022). Even though the SAS only ponders the RMSD and the number of superimposed residues, it gives a similar evolutionary signal compared to more elaborate scores such as the Qscore. In this work, the main clades as well as most of the inner branches are conserved; however, with the number of available structures and its limited diversity, it is not possible to set a timeframe to most of the scenarios here posited. An additional caveat to this technique is the difficulty calculating a statistical support for the results. Various alternatives have been tested addressing the issue; for instance, phylogenetic networks have proven to be successful solving conflicting evolutionary signals within the structural distance matrices (Lundin et al. 2012). On the other hand, proxies to the traditional bootstrap technique have also been adapted to structural phylogenies such as the use of hierarchical clustering (Suzuki and Shimodaira 2006), shape fluctuations derived from molecular dynamics simulations (Malik et al. 2020), and structural alphabets based on the amino acids’ tertiary interactions (Moi et al. 2023), all of which have shown encouraging results. There is no doubt that structural biology will continue to display the accelerated growth it has shown during this decade. The major improvements of tertiary structure predictors such as AlphaFold (Jumper et al. 2021) and RoseTTAFold (Baek et al 2021) are shifting the paradigms of biological and functional diversity in terms of tertiary structures. The aforementioned advances provide a very optimistic panorama regarding a more generalized implementation of tertiary structure-based phylogenies, which will, in turn, help unraveling blurry and intricatedeep evolutionary events.

## Supplementary Information

Below is the link to the electronic supplementary material.

Supplementary file1 (TIF 11182 kb) Supplementary Fig. 1 1-Q score-derived dendogram and phylogenetic network of proteins endowed with an NT-fold. **a** Structure-based dendogram and **b** structure-based phylogenetic network of proteins endowed with an NT-fold based on the 1-Q score. The name of the organism the structure was obtained from, and the name of the protein is written at each branch tip. The branches are colored according to the enzymatic function: cyan – template-independent RNA polymerases; blue—RNA binding; red—cellular signaling; chartreuse green—DNA repair polymerases; yellow—antibiotic-modifying enzymes; pink—specialized nucleotidyltransferases; olive green—protein binding; purple—replicative DNA-dependent DNA polymerases; turquoise blue—other functions. The symbol outside each enzyme corresponds to the cellular domain from which the structure was obtained: Bacteria—red star; Archaea—pink square; Eukaryotes—blue circle; Viruses—green triangle

Supplementary file2 (TIFF 532 kb) Supplementary Fig. 2 Structural superposition of archaeal CCA-adding enzyme (green) in the presence of tRNA and human oligoadenylate synthase (cyan) with double-stranded DNA bound

Supplementary file3 (TIFF 141 kb) Supplementary Fig. 3 A polyphaga mimivirus PolX homology-based tertiary structure prediction. The prediction was performed in the Phyre2 web server (Kelley et al. 2015). Domains are colored as follows: red—thumb; blue—palm; yellow—fingers; magenta—8kDa.

Supplementary file4 (TIFF 224 kb) Supplementary Fig. 4 Depiction of the synthetase NT fold (colored in blue). The side chains of the two conserved catalytic acidic residues are shown and colored in red. The positively charged wall is colored in green, the side chains of the residues with positive charge are shown. The long loop between strands 3 and 4 is colored in orange; the side chains of two conserved tyrosines are shown (Edited from PDB 6YXA)

Supplementary file5 (TIFF 229 kb) Supplementary Fig. 5 Representation of the Megavirus chilensis poly(A) polymerase. The catalytic NT fold is shown in blue, and the substrate binding helices are shown in green. The C-terminal domain is formed by a NT-fold (cyan) and two substrate binding helices (yellow) that lack the catalytic and the substrate binding residues (Edited from PDB 4P37)

Supplementary file6 (XLSX 13 kb)

Supplementary file7 (XLSX 87 kb)
