# Supplementary figures and images for: Structural and Evolutionary Analysis of Proteins Endowed with a Nucleotidyltransferase, or Non-canonical Palm, Catalytic Domain
Source: J Mol Evol. 2024 Sep 19;92(6):799–814. doi: 10.1007/s00239-024-10207-7 (PMC11646218; doi:10.1007/s00239-024-10207-7)

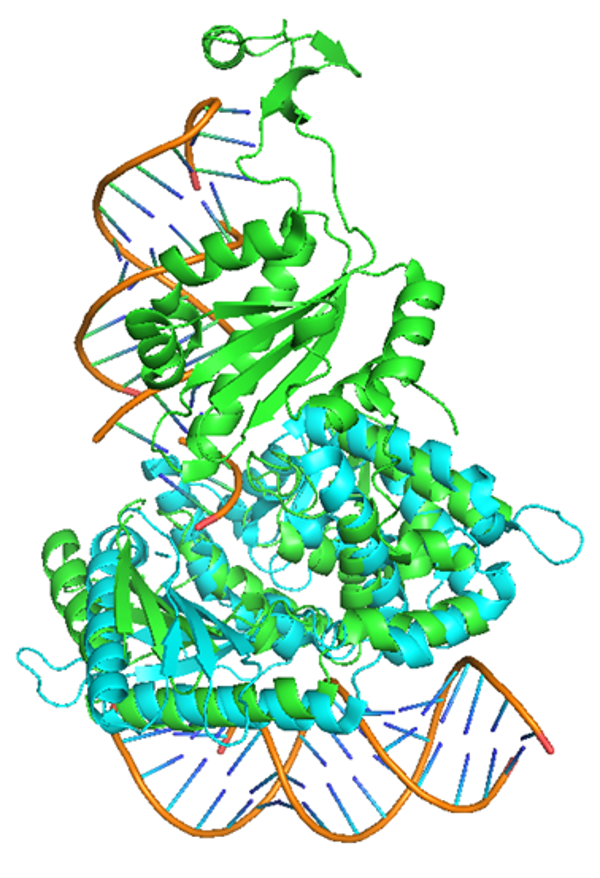

Supplement: Supplementary file 2 — Supplementary file2 (TIFF 532 kb) Supplementary Fig. 2 Structural superposition of archaeal CCA-adding enzyme (green) in the presence of tRNA and human oligoadenylate synthase (cyan) with double-stranded DNA bound [file 239_2024_10207_MOESM2_ESM.tiff]

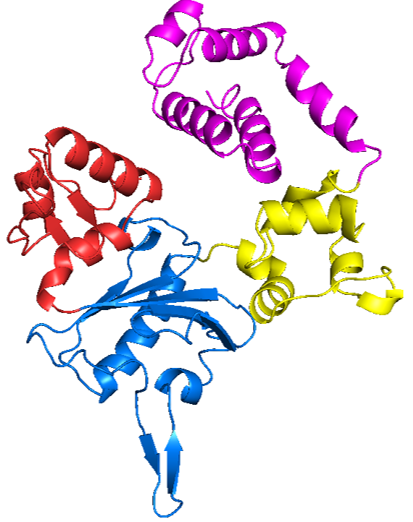

Supplement: Supplementary file 3 — Supplementary file3 (TIFF 141 kb) Supplementary Fig. 3 A polyphaga mimivirus PolX homology-based tertiary structure prediction. The prediction was performed in the Phyre2 web server (Kelley et al. 2015). Domains are colored as follows: red—thumb; blue—palm; yellow—fingers; magenta—8kDa. [file 239_2024_10207_MOESM3_ESM.tiff]

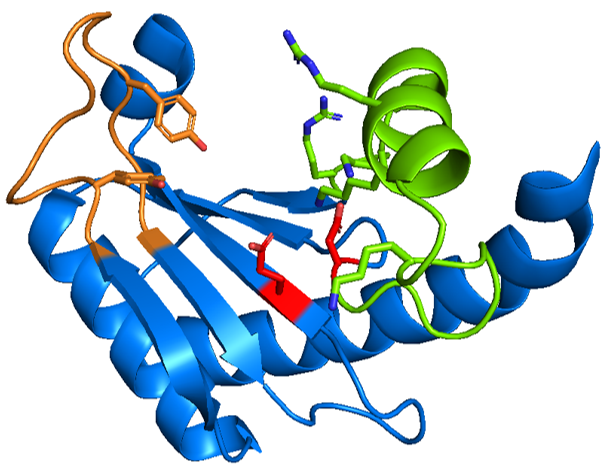

Supplement: Supplementary file 4 — Supplementary file4 (TIFF 224 kb) Supplementary Fig. 4 Depiction of the synthetase NT fold (colored in blue). The side chains of the two conserved catalytic acidic residues are shown and colored in red. The positively charged wall is colored in green, the side chains of the residues with positive charge are shown. The long loop between strands 3 and 4 is colored in orange; the side chains of two conserved tyrosines are shown (Edited from PDB 6YXA) [file 239_2024_10207_MOESM4_ESM.tiff]

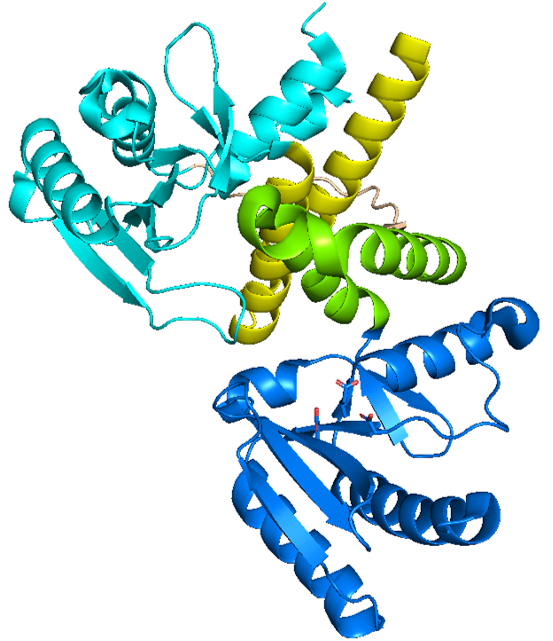

Supplement: Supplementary file 5 — Supplementary file5 (TIFF 229 kb) Supplementary Fig. 5 Representation of the Megavirus chilensis poly(A) polymerase. The catalytic NT fold is shown in blue, and the substrate binding helices are shown in green. The C-terminal domain is formed by a NT-fold (cyan) and two substrate binding helices (yellow) that lack the catalytic and the substrate binding residues (Edited from PDB 4P37) [file 239_2024_10207_MOESM5_ESM.tiff]
